# The Adhesion and Spoilage of *Shewanella putrefaciens* in Tilapia

**DOI:** 10.3390/foods11131913

**Published:** 2022-06-27

**Authors:** Wen Zhang, Ying Yu, Huihui He, Xucong Lv, Zhibin Liu, Li Ni

**Affiliations:** Institute of Food Science and Technology, College of Biological Science and Technology, Fuzhou University, Fuzhou 350108, China; zhangwen@fzu.edu.cn (W.Z.); 200820033@fzu.edu.cn (Y.Y.); duxing79842989@163.com (H.H.); xucong1154@163.com (X.L.); liuzhibin@fzu.edu.cn (Z.L.)

**Keywords:** adhesion, spoilage, *Shewanella putrefaciens*, microflora, volatile substance

## Abstract

*Shewanella putrefaciens* is a typical spoilage bacteria organism in seafood. The adhesion ability of three *S. putrefaciens* strains (HR-15, JR-18, HC-71) isolated from putrefied tilapia were evaluated by mucus adhesion in vitro and intestinal adhesion in vivo. The results of the spoilage of the inoculated fish fillets and spoilage of the refrigerated fish both showed that the adhesion ability of *S. putrefaciens* was positively correlated with the spoilage ability. High-throughput sequencing and GC-MS results showed that *S. putrefaciens* with high adhesion ability also significantly changed the intestinal flora of fish, causing an increase in the intestinal bacteria such as *Plesionomas*, *Macellibacteroides, Acinetobacter,* and *Legionella*, which then led to the increase in volatile substances such as low-grade aldehydes, alcohols, and ketones in the fish, serious fatty acid oxidation, and excitement of the fishy smell.

## 1. Introduction

Microbial degradative activity is one of the main causes of fish putrefaction [1,2]. *Shewanella* spp. is a group of Gram-negative bacteria with a high amine metabolism ability and is considered to be the dominant spoilage organism in seafood [1,3]. Feng et al. found that the microbial growth, TVB-N content, and K value of fish inoculated with *S. putrefaciens* increased faster than the control. The inoculation of *S. putrefaciens* accelerated the protein oxidation process of fish and promoted the breakage of their myogenic fibers, resulting in changes in the tertiary structure of fish proteins and a significant decrease in the moisture content of the samples [4]. Lakshmanan et al. found that *Shewanella* was the major amine-producing bacteria in shrimp during ice storage [5]. *Shewanella* spp. isolated from spoiled fish including *Shewanella oneidensis*, *Shewanella putrefaciens*, *Shewanella baltica*, and *Shewanella alga*, etc., showed spoilage potential [6,7]. It can transform trimethylamine-N-oxide (TMAO) to trimethylamine (TMA), resulting in fish putrefaction and deterioration [8]. *Shewanella* spp. degraded nitrogenous substances, proteins, and amino acids during low-temperature storage by modulating the amino acid transport and metabolism-related gene expression, which could produce amines, alcohols, aldehydes, ketones, and sulfides, reducing the sensory quality of fish [3].

The indicators characterizing microbial spoilage potential mainly include the total number of bacteria, trimethylamine (TMA), total volatile base nitrogen (TVB-N), K value, abilities to produce the enzyme, etc. [9]. Recent studies have also focused on the molecular mechanism of spoilage factors related to the regulation of its enzyme system and quorum sensing [3]. Our previous findings have shown that spoilage bacteria such as *Shewanella* adhere to fish through strong surface hydrophobicity and self-aggregation ability to enhance the adhesion to fish mucus and form a biofilm [10]. Our preliminary study showed that the adhesion ability of *Shewanella* spp. to the gill and intestine was higher than that to the skin, and the adhesion ability increased with the increase in the NaCl concentration [11]. These results suggest that high-salt concentration dependent adhesion is the reason why *Shewanella* can become the dominant spoilage bacteria of marine fish. We also found that the inhibition of spoilage bacteria adhesion by *B. subtilis* could reduce the spoilage effects [12].

This study further explored whether the differences in the ability of *Shewanella* to adhere to fish intestines would lead to the difference in spoilage ability. Three *S. putrefaciens* strains were screened, and their adhesion ability and spoilage ability in vitro and in vivo were compared. The role of the adhesion of spoilage bacteria in the spoilage of aquatic products was discussed based on the analysis of the bacterial community structure and volatile substances. The purpose was to reveal the role of the adhesion ability of *S. putrefaciens* in putrefied fish and to provide a theoretical basis for reducing the spoilage of aquatic products during storage.

## 2. Materials and Methods

### 2.1. Experimental Materials and Strains

Six *S. putrefaciens* strains (JR-6, HR-15, JR-18, JR-20, JC-48, and HC-71) were isolated from putrefied tilapia. The phylogenetic tree was identified by 16S rRNA and compared with the GenBank database, as shown in Supplementary Figure S1, and their sequence accession numbers are shown in Table 1.

### 2.2. Determination of the Adhesion Ability of S. putrefaciens In Vitro

#### 2.2.1. Bacteria Labeled with Fluorescein

*S. putrefaciens* was grown in TSB at 37 °C in a rotary shaker for 24 h. Bacterial cells were harvested by centrifugation at 8000 rpm for 10 min at 4 °C, washed twice with sterile PBS, and then re-suspended into 1 × 10^8^ CFU/mL bacterial suspension with 0.1 mol/L sterilized carbonate buffer with pH 9.2. FITC was added to the bacterial suspension with a final concentration of 0.2 mg/mL. The bacteria suspension was incubated at 100 rpm for 2 h at 30 °C in the dark, then centrifuged at 4000 rpm for 15 min. The cells were washed twice with sterile PBS to remove the unincorporated FITC, then resuspended in sterile PBS solution with the final concentration of the bacterial suspension of 1 × 10^8^ CFU/mL [13].

#### 2.2.2. Determination of Adhesion In Vitro

Mucus was prepared using the method published by Chen et al. [14] with slight modification. The adhesion rate was determined according to Zhang et al. [12].The adhesion rate was calculated using Equation (1).

(1) $$\mathrm{Adhesion}\;\mathrm{rate}\%=\frac{A_{2}-A_{0}}{A_{1}}\times 100$$

where *A*_1_ and *A*_2_ are the fluorescence intensities of *S*. *putrefaciens* after adhesion and pure *S*. *putrefaciens* suspension, respectively; *A*_0_ is the fluorescence intensity of the blank group.

### 2.3. Determination of Spoilage Ability

#### 2.3.1. Preparation and Inoculation of Sterile Fish Fillets

The fresh tilapias were washed with sterile water and the scales, gills, and guts were removed. Then, the water was removed with absorbent paper and sprayed with 75% alcohol. The skin of the fish was removed with a sterile scalpel, and the dorsal muscles of the fish were cut into fillets with a mass of 2.0 ± 0.1 g. The fish fillets were sprayed with 75% alcohol and placed on an ultra-clean bench for 20 min of UV irradiation.

Sterile fish fillets were soaked in a 40 mL bacterial suspension of *S. putrefaciens* (adjusted to OD600 = 1.0 ± 0.05, approx. 1 × 10^8^ CFU/mL) for 30 s, drained, put into a sterile homogeneous bag (approx. 10.0 g per bag), and then stored at 4 °C with non-inoculated fish fillets as the blank control. The total bacterial count and TVB-N in the fish fillets were determined on days 1, 3, and 5 to compare the spoilage ability of *S. putrefaciens*.

#### 2.3.2. Total Bacteria in Fish Fillets

We weighed 2.0 g of the fish fillets prepared from Section 2.3.1, put them into sterile homogenization bags added with 10 mL normal saline, fully homogenized them, and the diluted them with ten times gradient. We then took 100 µL of the diluent and inoculated it on the TSA medium plate, incubated it at 30 °C for 24 h, then calculated the number of colonies in parallel three times for each dilution.

#### 2.3.3. Determination of Total Volatile Basic Nitrogen (TVB-N)

TVB-N was determined following the method described by Zhang et al. [12], and calculated using Equation (2):

(2) $$X=\frac{\left(V_{2}-V_{1}\right)\times c\times 14}{m}\times 100$$

where V_1_ and V_2_ are the volume (mL) for the sample and reagent blank; c is the concentration of HCl (mol/L); and m is the mass of the sample (g).

#### 2.3.4. The Yield Factor *Y*_TVB-N/CFU_ of TVB-N

Referring to the method of Dalgaard P. [15], the amount of spoilage metabolites produced per unit of total bacteria at the end of refrigerated storage was taken as the quantitative index of the bacterial spoilage ability *Y*_TMA/CFU_ algorithm, and the yield factor *Y*_TVB-N/CFU_ was calculated with spoilage metabolite TVB-N [16]. The calculation formula is as follows:

(3) $$Y_{\text{TVB-N/CFU}}=\frac{{\left(\text{TVB-N}\right)}_{s}-\;△\text{TVB-N}}{N_{s}{\;-\;N}_{0}}$$

where: *N*_0_ and *N_s_* are the initial numbers of colonies and the total number of colonies during chilled storage/(CFU/g); △TVB-N and (TVB-N)s are the TVB-N content/(mg/g) at the initial and endpoints of refrigeration, respectively.

### 2.4. In Vivo Test

#### 2.4.1. Feeding Experiment of Tilapia

The healthy tilapia juveniles, with an average weight of 30.00 ± 5.00 g, were provided by the Fuzhou Aquaculture Institute. Two hundred and forty tilapia were randomly divided into the control group and the *S*. *putrefaciens* treated groups (HR-15 group, JR-18 group, and HC-71 group), with three replicates in each group and 20 tilapia in each replicate. The fish of the same treatment group were put into a 100 L plastic bucket with 50 L of overnight dechlorinated fresh water, in the temperature range of 25–30 °C. The air pumps were used to add oxygen. The basic fish feed was purchased from Fuzhou Haima Feed Co., Ltd. (Fuzhou, China) and its main ingredients included flour, fish meal, fish oil, soybean meal, and many vitamins and minerals. Feed was supplied twice a day (09:30 and 17:30 h). After feeding (10:00), approximately 50% of the cultivation water was changed daily, unconsumed feed and fish feces were purged, then 5 mL of the bacterial suspension (1.0 × 10^8^ CFU/mL) was added to 50 L of the aquaculture water of the treatment groups, respectively. The feeding test lasted for 14 days including 7 days of the adaptation period (without bacterial suspension) and 7 days of the test period. No fish died during the test period.

#### 2.4.2. Sample Collection and Processing

At the end of the experimental period, tilapia fasted for one day. After the young fish were killed, they were grouped into sterile homogeneous bags and stored at 4 °C. The experimental fish were randomly taken from each group, refrigerated for 0 and 6 days, dissected with a sterile scalpel, and then the intestinal tract and back flesh of the tilapia were taken out. The removed intestine was repeatedly washed with sterile water to remove the excess fat, then 0.2 g of the intestinal content and 0.5 g of the flesh were weighed, and placed in a 2 mL centrifuge tube for DNA extraction. In addition, 10.0 g of fish flesh was placed in a sterile homogeneous bag for the determination of TVB-N using the method in Section 2.3.3. The yield factor *Y*_TVB-N/CFU_ of the TVB-N was calculated with reference to Section 2.3.4.

### 2.5. Determination of Volatile Flavor Compounds by GC-MS

A total of 2.0 g refrigerated fish sample was put into a 15 mL headspace bottle with a micro stirrer, 5 mL of the saturated NaCl solution was added, then it was covered and sealed, placed in a 60 °C water bath for balance for 3 min, then the aged (250 °C, 0.5 h) extraction head was inserted into the silicone rubber bottle pad of the headspace bottle, about 1 cm above the liquid level, left to absorb in the headspace for 0.5 h, then quickly taken out and inserted into the GC-MS (7890-B/5977A, Agilent Technologies Inc., Santa Clara, CA, USA) injection port for desorption for 3 min, and the determination was repeated for three times in each group [17].

In the extraction bottle, 200 μL of 2,4,6-trimethylpyridine (2 mg/L) was added as the internal standard. The concentration of the compound was calculated based on the ratio of the peak areas of the flavor compounds, as per Equation (4).

(4) $$\mathrm{Volatile}\;\mathrm{component}\;\mathrm{concentration}\left(\mu g/g\right)=\frac{\frac{\mathrm{Ai}}{A}\times 0.4}{2}$$

where Ai and A are the peak areas of volatile component i and the internal standard, respectively, 0.4 is the amount of TMP added (μg), and 2 is the mass of the tested sample (g).

### 2.6. Detection of Specific Bacteria in the Intestines and Flesh by Quantitative Real-Time PCR (qPCR)

The extraction of the microbial DNA from the tilapia flesh and intestine was conducted by referring to the procedure on the DNA extraction kits (Fecal Genomic DNA Extraction Kit and Bacterial Genomic DNA Extraction Kit (Tiangen, Beijing, China). The extracted DNA was detected by the SYBR Green dye method. The specific primer sequence is shown in Table 2, and was synthesized by Shanghai Shenggong Co., Ltd. (Shanghai, China). The specificity of the primers was verified, and the standard curves of the bacterial concentration and Ct value were drawn. The bacterial concentration was obtained by the plate counting method, and qPCR established the linear relationship between the Ct value and bacterial concentration. The changes in the target bacteria during storage were determined by measuring the Ct values of the total bacteria and *Shewanella* in the fish intestines.

The PCR was performed as follows: 30 s at 95 °C; 45 cycles of 5 s at 95 °C, 30 s at 60 °C, and 30 s at 72 °C. The solubility curve analysis procedure was as follows: first hold at 72 °C for 15 s, followed by 60 °C for 1 min, then ramp up to 95 °C for 15 s.

### 2.7. Analysis of Changes of Intestinal Microflora in Tilapia by High-Throughput Sequencing

Using the extracted fish gut genomic DNA as a template, specific primers (bacterial primers 341-F (50-CCT AYG GGR BGC ASC AG-30) and 806-R (50-GGA CTA CNN GGG TAT CTA AT-30) with barcodes were synthesized according to the V3–V4 region sequences for PCR amplification. The specific steps for high-throughput sequencing were according to Zhang et al. [12].

### 2.8. Statistical Analysis

All assays were conducted in triplicate independently. The adhesion rate, TVB-N value, and TVC were expressed as the mean ± standard deviation, plotted by GraphPad Prism 8.0 and analyzed by ANOVA with the LSD multiple comparison post hoc test using SPSS 25.0 software. The significance of difference was *p* < 0.05. The PCA score plots was plotted by SIMCA14.1. The relationship between the functional microbiota and flavor formation was elucidated by OPLS modeling. The correlation network analysis plot was plotted by Cytoscape.

## 3. Results

### 3.1. Adhesion and Spoilage of S. putrefaciens In Vitro

#### 3.1.1. Adhesion Rate of *S. putrefaciens* in Different Mucus

**Figure 1:**
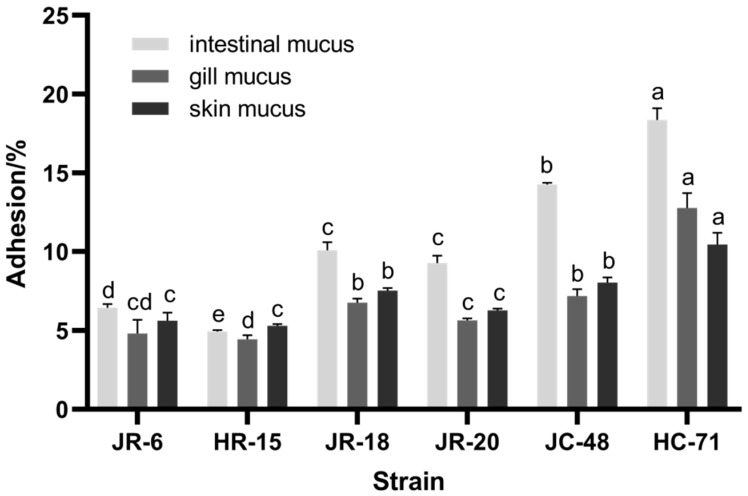
The adhesion rate of six *S. putrefaciens* strains (JR-6, HR-15, JR-18, JR-20, JC-48, and HC-71) in different mucus. Different lowercase letters indicate significant differences at the 0.05 level (*p* < 0.05).

The adhesion rate of six *S. putrefaciens* strains on the surface, gill, and intestinal mucus of tilapia were compared. As shown in Figure 1, the adhesion ability of *S*. *putrefaciens* to the intestinal mucus was stronger than that of the gill mucus and skin mucus (*p* < 0.05). Three *S*. *putrefaciens* strains (HC-71, JR-18, and HR-15) were selected as representatives of the high, medium, and low adhesion strains for subsequent in vitro spoilage ability tests and in vivo tests. Larsen [13] found that the chemotaxis of *Vibrio anguillarum* to the intestinal mucus of rainbow trout was significantly higher than that of gill mucus, which was similar to the adhesion in our study results.

#### 3.1.2. Analysis of Spoilage Ability In Vitro

**Figure 2:**
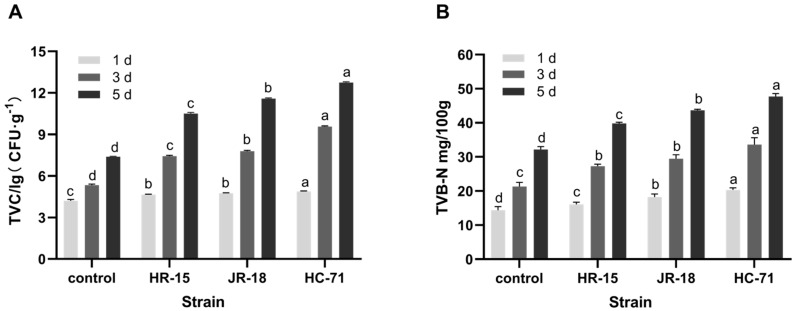
The changes in the total viable counts (TVC) (**A**) and TVB-N (**B**) of the fish fillets inoculated with three *S*. *putrefaciens* strains (HR-15, JR-18, and HC-71) stored at 4 °C. Different lowercase letters indicate significant differences at the 0.05 level (*p* < 0.05).

The total number of bacteria in fish fillets inoculated with *S*. *putrefaciens* increased rapidly, which quickly led to spoilage (*p* < 0.05). As shown in Figure 2A, the HC-71 group was up to 5.52 × 10^12^ CFU/g on the fifth day, followed by the JR-18 group at 3.89 × 10^11^ CFU/g, and the minimum number of colonies in the HR-15 group was 3.21 × 10^10^ CFU/g. These substances affect the flavor and quality of food and are related to the shelf life of aquatic products [5,18]. As shown in Figure 2B, the TVB-N of fish fillets increased with the extension of refrigeration time. After three days of chilled storage, the TVB-N in the HC-71 group exceeded 25 mg/100 g. The increasing trend of TVB-N was the same as that of the total bacteria, that is, the HC-71 group was higher than the JR-18 group and the HR-15 group.

The yield factor of TVB-N is related to the ability of bacteria to break down proteins and the number of bacteria [19]. Therefore, *Y*_TVB-N/CFU_, the yield factor of the TVB-N metabolized by spoilage microorganisms, was used as the evaluation index of the spoilage ability of spoilage bacteria to fish in vitro [15]. As shown in Table 3, among the three strains of *S. putrefaciens*, the TVB-N yield factor of the HR-15 group was the highest, which was 7.39 × 10^−10^ mg/CFU, followed by the JR-18 and the HC-71 (4.96 × 10^−12^ mg/CFU). Although the HC-71 produced the most TVB-N, its total number of bacteria was also the highest, so the HC-71 strain did not show a high spoilage ability in vitro.

### 3.2. Adhesion and Spoilage of S. putrefaciens In Vivo

Unlike the putrefaction of fish fillets in vitro, the spoilage by spoilage bacteria in vivo is not only affected by the spoilage ability [18], but depends on whether the bacteria can adhere to the fish. Therefore, the in vivo feeding test was used to explore the spoilage of *S*. *putrefaciens* with different adhesion abilities to fish.

**Figure 3:**
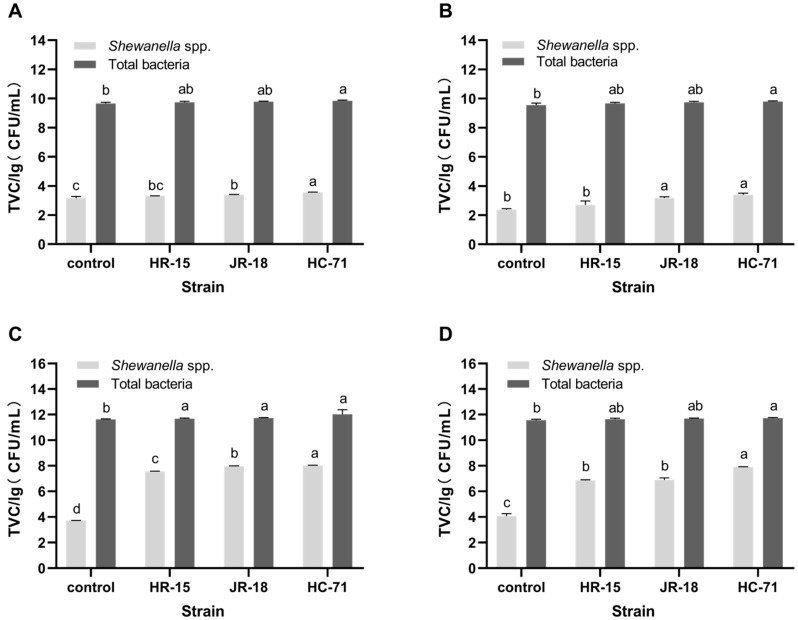
The qPCR analysis of the bacterial growth in the intestine and flesh of tilapia during storage at 4 °C. *Shewanella* spp. and total bacteria content in the intestine (**A**) and flesh (**B**) after 0 days of refrigeration; *Shewanella* spp. and total bacteria content in the intestine (**C**) and flesh (**D**) after 6 days of refrigeration. Different lowercase letters indicate significant differences at the 0.05 level (*p* < 0.05).

The total number of bacteria and the content of *Shewanella* spp. in the tilapia intestines and flesh were determined by qPCR (*p* < 0.05). On day 0, there was no significant difference in the total number of bacteria in each group, except that the HC-71 group was higher than that of the control (Figure 3A). The number of *Shewanella* spp. in the intestine of the HC-71 and JR-18 groups with strong adhesion ability was significantly higher than that of HR-15 and the control (Figure 3A). As Figure 3A,B shows, the total amount of bacteria in the fish flesh was slightly lower than that in the fish intestines, the number of *Shewanella* spp. in the fish flesh was significantly lower than that in fish intestines, and the trend of difference between the groups was consistent with that in the fish intestines.

On the sixth day of refrigeration, the bacteria in the fish intestines and flesh proliferated. As shown in Figure 3C,D, the total number of bacteria in the treated group with the addition of *Shewanella* spp. was slightly higher than that in the control group, and there was no significant difference between the treatment groups. It was shown that *Shewanella* spp. spread significantly during storage. *Shewanella* spp. was significantly higher in the treated group than in the control group, especially in HC-71. The in vivo test also showed that the colonization ability of *Shewanella* spp. with strong adhesion ability in the fish intestine was stronger, and the addition of *Shewanella* spp. had little effect on the total number of bacteria, but *Shewanella* spp. had obvious growth advantages in the later stage, and the relative proportion of strain with a high adhesion ability was significantly higher.

**Figure 4:**
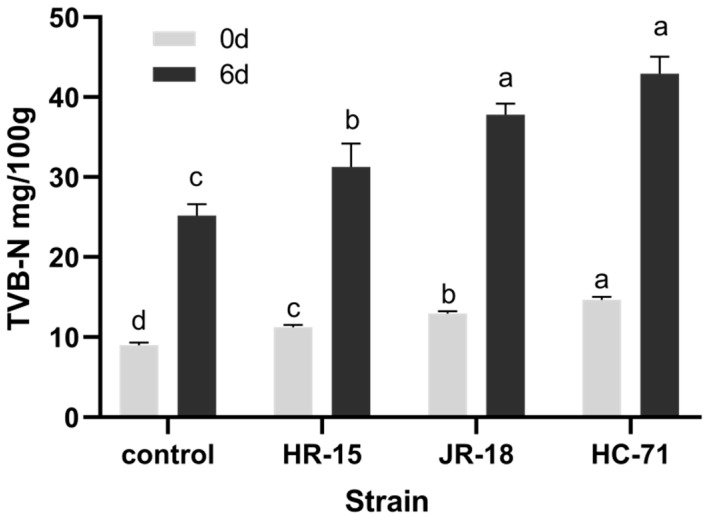
The TVB-N value of tilapia inoculated with different *S*. *putrefaciens* strains (HR-15, JR-18, and HC-71) and stored at 4 °C. Different lowercase letters indicate significant differences at the 0.05 level (*p* < 0.05).

As shown in Figure 4, the TVB-N in the treated groups with the addition of *Shewanella* spp. was significantly higher than that in the control group, and the TVB-N also increased with the enhancement in the strain adhesion. It showed that the colonization of spoilage bacteria in fish would accelerate the degree of spoilage. On day 0, the freshness of tilapia with the high adhesion ability strain HC-71 and medium adhesion ability strain JR-18 was only in the secondary freshness range. After six days of refrigerated storage, the TVB-N of strain HC-71 and strain JR-18 with high adhesion ability had significantly exceeded the shelf endpoint by 30 mg/100 g. The sanitary standards for fresh and frozen animal aquatic products (GB 2733-2005, China) state that a TVB-N of a sample exceeding 30 mg/100 g is considered as spoiled [20].

The yield factor *Y*_TVB-N/CFU_ of the TVB-N was calculated to evaluate the spoilage ability of the three strains of *S*. *putrefaciens* in vivo. As shown in Table 4, the highest yield factor of TVB-N in the treated group of high adhesion bacterium HC-71 was 5.49 × 10^−11^ mg/CFU. The spoilage ability showed a positive correlation with the adhesion ability. Unlike the spoilage ability in vitro, the spoilage ability (*Y*) of a single colony in vivo was positively correlated with the adhesion ability. Since the spoilage ability of the bacteria itself did not change, it was speculated that the increase in *Y* was due to the ability of other bacteria to cause spoilage and produce TVB-N.

### 3.3. Effects of S. putrefaciens on the Structural Diversity of Tilapia Flora and Fish Flavor

#### 3.3.1. Intestinal Flora Composition Based on High-Throughput Sequencing Results

**Figure 5:**
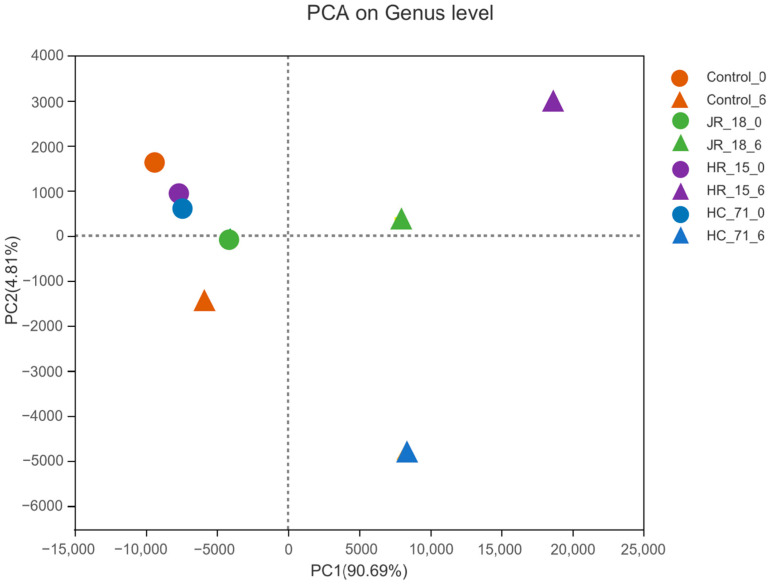
The principal component analysis on the genus level of the intestinal flora of tilapia inoculated with different *S*. *putrefaciens* strains (HR-15, JR-18, and HC-71).

The intestinal flora of the tilapia samples stored for 0 and 6 days was sequenced by high-throughput sequencing. The composition of intestinal flora in each group was analyzed by principal component PCA analysis. The PCA analysis showed that the samples on day 0 were concentrated and had a relatively similar flora composition (Figure 5). After 6 days of storage, the difference in flora composition was obvious. HC-71 and JR-18 with a high adhesion ability were close, located between the positive axis of PC1 and the negative axis of PC2; HR-15 was located between the positive axis of PC1 and PC2; while the similarity of con0 and con6 in the control group were close, indicating that the addition of *S*. *putrefaciens* significantly affected the flora composition and had a certain relationship with the adhesion ability of the strains.

**Figure 6:**
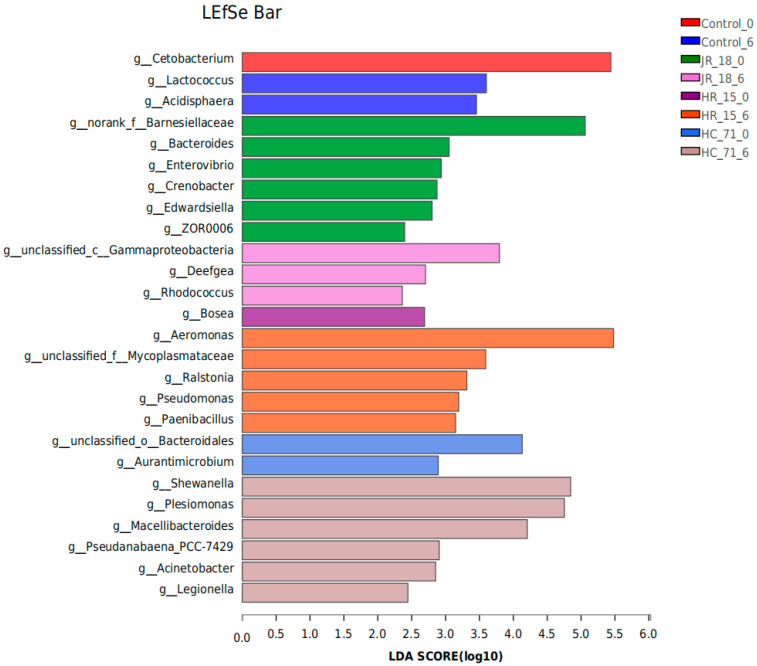
The LEfSe analysis on the genus level of the intestinal flora of the tilapia inoculated with different *S*. *putrefaciens* strains (HR-15, JR-18, and HC-71).The higher the LDA score, the more obvious the impact of the species with differences between groups.

Furthermore, LEfSe analysis showed the species with significant differences between groups (Figure 6). The higher the LDA score, the more obvious the impact of the species with differences between groups. On day 0 of refrigerated storage, *Cetobacterium*, *Bosea*, *Barnesiellaceae*, and *Bacteroidales* were differentially dominant bacteria. *Cetobacterium* is a common bacterium in fish. *Barnesiellaceae* mostly comes from water pollution including *Bacteroidales*, a common pathogen of fish [21]. On day 6 of storage, the differential bacteria in the control group changed into *Lactococcus* and *Acidisphaera*. The number of differential species in the treated group increased significantly. Among them, the differential species in the highly adhered HC-71 group evolved into *Shewanella*, *Plesionomas*, *Macellibacteroides*, *Acinetobacter*, and *Legionella*. The results showed that highly adhered *Shewanella* was easier to colonize and became a dominant strain in the intestinal flora, and its addition reduced the host immunity, which increased the susceptibility to pathogens such as skin lesions.

#### 3.3.2. Volatile Components in Putrefied Fish

The PCA analysis of the volatile components in fish flesh is shown in Figure 6. The volatile components in each sample were similar at day 0, but the flavor changed greatly after 6 days of refrigeration. After 6 days of spoilage, the content and types of esters in the *S*. *putrefaciens* treated group increased. Esters are the products of the lipid oxidation of fish flesh, and the results indicated that *S*. *putrefaciens* would lead to frequent bacterial metabolic activities and increase the bad flavor of the fish flesh.

**Figure 7:**
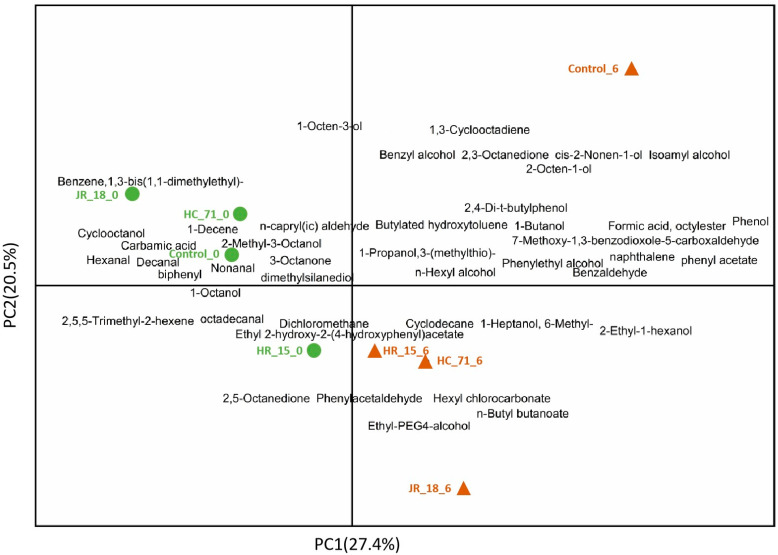
The PCA score plots of the volatile compounds in the different treated groups. The control group (Con_0: refrigerate for zero days; Con_6: refrigerate for six days) was fed with common feed, and the experimental groups (HR-15 (HR-15_0, HR-15_6), JR-18 (JR-18_0, JR-18_6), and HC-71 (HC-71_0, HC-71_6)) were fed with the supplemented *S. putrefaciens* feed.

As shown in Figure 7, the flavor composition of the control group and treated group changed on day 6. On day 6 of the control group, the lower alcohol and aldehyde volatile substances such as 1-octene-3-alcohol, 2,3-octanedione, and 2-octene-1-alcohol were increased. 1-Octene-3-alcohol has a fishy, earthy, and mushroom smell. Through the study of the volatile substances of bighead carp, Zhao Qingxi et al. [22] believe that 2,3-octanedione is related to the generation of a fishy smell, which is the decomposition of polyunsaturated fatty acids (arachidonic acid) caused by lipoxygenase or the reduction in carbonyl compounds.

The addition of *S. putrefaciens* increased the 2-ethyl-1-hexanol, 2,5-octanedione, 7-methoxy-1,3-benzodioxole-5-carboxaldehyde, and 1-heptanol, 6-methyl-. 2-Ethyl-1-hexanol has a fat and burning smell; 1-heptanol, 6-methyl-, and 2,5-octanedione have a special fishy smell and a fat smell of metal. 7-Methoxy-1,3-benzodioxole-5-carboxaldehyde is a aldehyde, and its ROVA value is only 0.09 [23]. A large number of studies have shown that fishy substances are mainly composed of volatile substances such as small molecules of aldehydes, ketones, alcohols, and sulfur compounds [24]. Aldehydes are the products of the further degradation of hydroperoxide, the primary product of unsaturated fatty acid oxidation [25]. Their threshold is generally very low, which is related to the smell of grass and fish in aquatic products [26]. When they exist in trace amounts, they still make a great contribution to the overall odor characteristics of fish.

#### 3.3.3. Correlation Analysis between Intestinal Flora and Volatile Flavor Compounds

**Figure 8:**
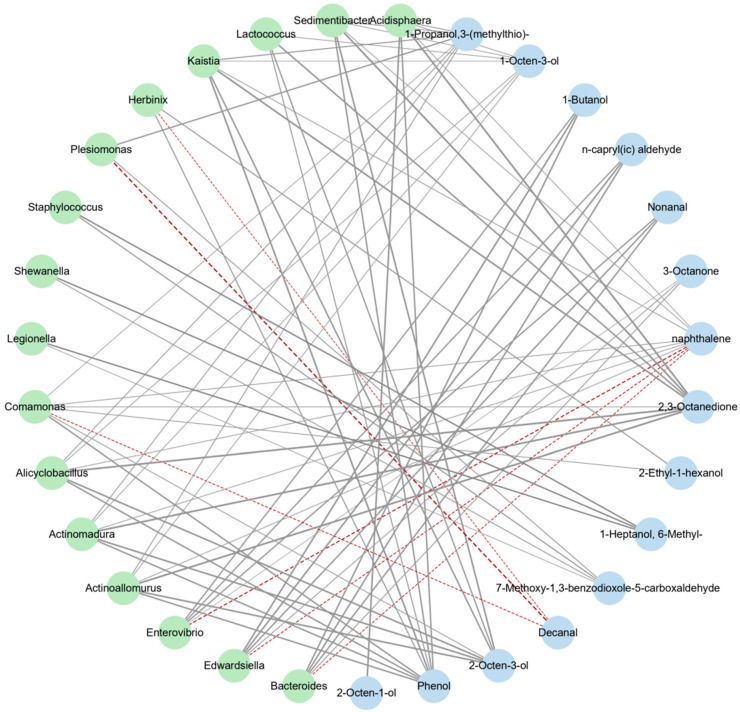
The correlation network analysis plot of the intestinal flora and volatile flavor compounds. Negative correlation (red dotted line). Positive correlation (gray solid line).

The volatile compounds (excluding alkanes) produced in the process of spoilage were combined with 16 strains (|ρ| > 0.6), and the correlation between the flora and volatile compounds in the process of fish putrefaction was studied by OPLS analysis. As shown in Figure 8, the addition of *S. putrefaciens* resulted in the increase in *Shewanella*, *Plesionomas*, *Legionella*, and other strains, resulting in the generation of lower alcohol and aldehyde volatile substances such as 7-methoxy-1,3-benzodioxole-5-carboxaldehyde and 1-heptanol, 6-methyl-. In general, most of the volatile substances produced exacerbate the unpleasant smell of fish such as the smell of grass, soil, fat oxidation, and rancidity.

## 4. Discussion

Bacterial adhesion to the mucosal surface appears to be a prerequisite for successful infection, and therefore more work is needed to focus on adhesion, which was considered to be the main virulence factor. Exopolymers and flagella play an important role in establishing the initial interactions with the mucosal surfaces or cells [27]. Some studies have found that the hydrophobicity and self-aggregation were closely related to the biofilm formation, and adhesion was the first step of biofilm formation [28]. Adhesion is important for pathogenic bacteria, but also spoilage bacteria. Our study tried to reveal that adhesion is an important spoilage factor of spoilage bacteria.

Three strains of *S. putrefaciens* with high, medium, and low adhesion rates, HC-71, JR-18, and HR-15, respectively, were selected to inoculate the fish fillets and compare their in vitro spoilage ability. The results showed that there was a significant positive correlation between the adhesion ability of *S. putrefaciens* and spoilage ability. TVB-N mainly includes trimethylamine, dimethylamine, ammonia, and other nitrogen-containing substances formed by the protein decomposition of the fish due to the action of enzymes and bacteria [29]. However, it is not absolute to use the total number of colonies and TVB-N to reflect the spoilage capacity of a strain, and the spoilage capacity of a strain could be comprehensively reflected by calculating the yield factor of spoilage. By calculating *Y*_TVB-N/CFU_, HC-71 with the high adhesion capacity had the lowest yield factor *Y*_TVB-N/CFU_. Since the strong adhesion indicated that the biofilm formation ability was also strong [10], this made the total number of colonies of HC-71 large, resulting in the highest TVB-N, but the spoilage ability of a single bacterium was not the highest.

In the fish in vivo adhesion test, combined with high-throughput sequencing and qPCR technology, the growth and reproduction of the target strain in the intestine were analyzed. The results showed that HC-71 with high adhesion was the highest in the intestine and fish muscles, and HR-15 with low adhesion was the lowest, but it could not cause significant changes in the total number of colonies. The TVB-N results showed that HC-71 with the high adhesion ability also had a high spoilage ability in vivo. The yield factor *Y*_TVB-N/CFU_ of HC-71 was the highest, but it was relatively low in vitro. It was speculated that HC-71 might regulate other spoilage bacteria through other ways such as quorum sensing, which was validated in the results of the high-throughput and fish flavor analysis. The ability of highly adherent *S. putrefaciens* to colonize the intestine was stronger, which will significantly change the abundance of flora and species differences. The addition of *S. putrefaciens* not only increased the proportion of *S.* spp. in the fish intestinal tract but also caused the breeding of Aeromonas, which is a common pathogen in aquaculture. At the same time, the proportion of *Pseudomonas* also increased, which is also a common dominant spoilage bacterium of aquatic products. It has a strong ability to produce putrefaction products such as ammonia and TVB-N, which can accelerate the spoilage effect [30].

Once adhesion occurs, organisms may experience specific molecular changes such as changes in the fish flavor. Ketones may be produced by fatty acid oxidation, amino acid degradation, and the interaction between the two products. They have a certain enhancement effect on the fishy smell, but have a high threshold and low content [31]. Direct aldehydes such as hexanal, heptanaldehyde, octanal, nonanal, and decanal can be formed rapidly through lipid oxidation, which is related to the grass smell, fat smell, and Khara taste (oil smell, fat oxidation, and rancidity smell) of aquatic products, among which phenylacetaldehyde has a strong earthy smell. It is generally believed that the threshold of aldehydes is low, but they have a strong odor superposition effect. When they exist in trace amounts, they still make a great contribution to the overall odor characteristics of fish. Alcohols generally come from the reduction of sugars, amino acids, and aldehydes.

Adhesion led to the formation of biofilms including the release of EPS. EPS has been found as a polymer that directly affects the interaction between bacteria and their hosts such as the adhesion of bacteria to the gut epithelium, their effect on immune response, and intestinal microbiota [32]. The presence of intestinal probiotics creates a good microecological environment for the host, while fish symbiosis provides the conditions for the growth and reproduction of the intestinal microflora. The intestinal microflora and the host are interdependent and mutually regulated [33]. However, the presence of spoilage bacteria can destroy this good microecological environment. We surmise that the intestinal flora is one of the important factors affecting the physiological characteristics of fish. *S*. *putrefaciens* with a high adhesion colonized the intestine and became the dominant bacteria. The disturbance of the intestinal flora of fish caused by *S*. *putrefaciens* leads to the dominance of other intestinal floras that are not conducive to the growth of fish. It is also possible that the presence of *S*. *putrefaciens* leads to the reduction in intestinal oxygen, resulting in the growth of anaerobic bacteria. Immune function host and intestinal bacteria are complex systems that interact with each other, especially the natural intestinal flora, which has become an indispensable part of the fish. Once the immune function of the fish is destroyed (such as the colonization of *S*. *putrefaciens* and other spoilage bacteria), the immune function of the fish cannot resist other spoilage bacteria or pathogens, so it is easier for them to be colonized. Gaulke et al. found that the interaction of these intestinal floras affected the growth of parasites and maintained the internal homeostasis of fish [34]. However, changes in the fish environment such as water temperature, food types, and viral and bacterial infections can disrupt the balance of the intestinal flora and may lead to the abnormal growth of pathogens, which has been shown to be closely associated with the development of fish diseases [35,36].

## 5. Conclusions

There was a correlation between the intestinal adhesion ability of *S. putrefaciens* and the putrefaction ability of fish flesh. The intestinal adhesion ability of *Shewanella* spp. can be used as one of the indicators of its putrefaction ability in fish. The TVB-N content and the total number of colonies of tilapia flesh fed with high adhesion *S*. *putrefaciens* were higher, the total number of volatile components increased, and the low-grade aldehydes and alcohols increased, the fat oxidation was serious, and the putrefaction flavor was aggravated. The addition of *S*. *putrefaciens* resulted in the increase in *Shewanella*, *Plesionomas*, *Legionella*, and other strains, resulting in the generation of lower alcohol and aldehyde volatile substances such as 7-methoxy-1,3-benzodioxole-5-carboxaldehyde and 1-heptanol, 6-methyl-. The correlation between the intestinal flora and volatile components proves that the feeding of strong adhesion *S*. *putrefaciens* affects the intestinal flora of fish, and that the change of intestinal flora further affects the spoilage of the whole fish and the flavor of the fish flesh.

## Figures and Tables

**Table 1:** The GenBank accession numbers of the nucleotide sequence of the experimental strain.

Strain	GenBank Accession Number
*S*. *putrefaciens* JR-6	ON505449
*S*. *putrefaciens* HR-15	ON505450
*S*. *putrefaciens* JR-18	ON505451
*S*. *putrefaciens* JR-20	ON505452
*S*. *putrefaciens* JC-48	ON505453
*S*. *putrefaciens* HC-71	ON505454

**Table 2:** The specific primer sequence of the strain.

Strain	Primer	Sequence (5′→3′)
Total bacteria	27-F	AGAGTTTGATCCTGGCTCAG
	1429-R	GGTTACCTTGTTACGACTT
*Shewanella* spp.	She3-F	GGAAACCCTGATGCAGCCAT
	She3-R	AAGCTGCTACCTTTCCTCCCT

**Table 3:** The yield factors of the TVB-N of the fish fillets inoculated with different *S*. *putrefaciens* strains (HR-15, JR-18, and HC-71) during chilled storage at 4 °C.

*S. putrefaciens*	The Total Number of Colonies/(CFU/g)		TVB-N/(mg/100 g)		*Y*_TVB-N/CFU_/(mgTVB-N/CFU)
	*N*_0_	*N_s_*	TVB-N_0_	TVB-N_s_	
Control	1.61 × 10^4^	2.45 × 10^7^	14.37	32.15	-
HR-15	4.42 × 10^4^	3.21 × 10^10^	16.10	39.81	1.85 × 10^−10^
JR-18	5.56 × 10^4^	3.89 × 10^11^	18.25	43.68	1.97 × 10^−11^
HC-71	7.50 × 10^4^	5.52 × 10^12^	20.35	47.74	1.74 × 10^−12^

**Table 4:** The yield factors of the TVB-N of the tilapia flesh inoculated with different *S*. *putrefaciens* strains (HR-15, JR-18, and HC-71) during refrigerated storage at 4 °C.

*S. putrefaciens*	The Total Number of Colonies/(CFU/g)		TVB-N/(mg/100 g)		*Y*_TVB-N/CFU_/(mgTVB-N/CFU)
	*N*_0_	*N_s_*	TVB-N_0_	TVB-N_s_	
Control	3.54 × 10^9^	3.70 × 10^11^	8.96	25.20	-
HR-15	4.63 × 10^9^	4.29 × 10^11^	11.25	31.27	6.53 × 10^−11^
JR-18	5.38 × 10^9^	4.87 × 10^11^	12.93	37.80	7.49 × 10^−11^
HC-71	6.21 × 10^9^	5.22 × 10^11^	14.65	42.93	8.06 × 10^−11^

## Data Availability

The data presented in this study are available on request from the corresponding author.

## Supplementary Materials

The following are available online at www.mdpi.com/article/10.3390/foods11131913/s1: Figure S1: The phylogenetic tree analysis plot of 18 bacterial strains isolated from the tilapia and their living environment; Table S1: The determination of the volatile flavor compounds in the fish flesh after storage for zero and six days by GC-MS.
