# Supplementary material for: The Adhesion and Spoilage of Shewanella putrefaciens in Tilapia
Source: Foods. 2022 Jun 27;11(13):1913. doi: 10.3390/foods11131913 (PMC9266031; doi:10.3390/foods11131913)
Supplement: Supplementary file 1 [file foods-11-01913-s001.zip › foods-1750663-supplementary.pdf]

foods

MDPI

Supplementary Materials

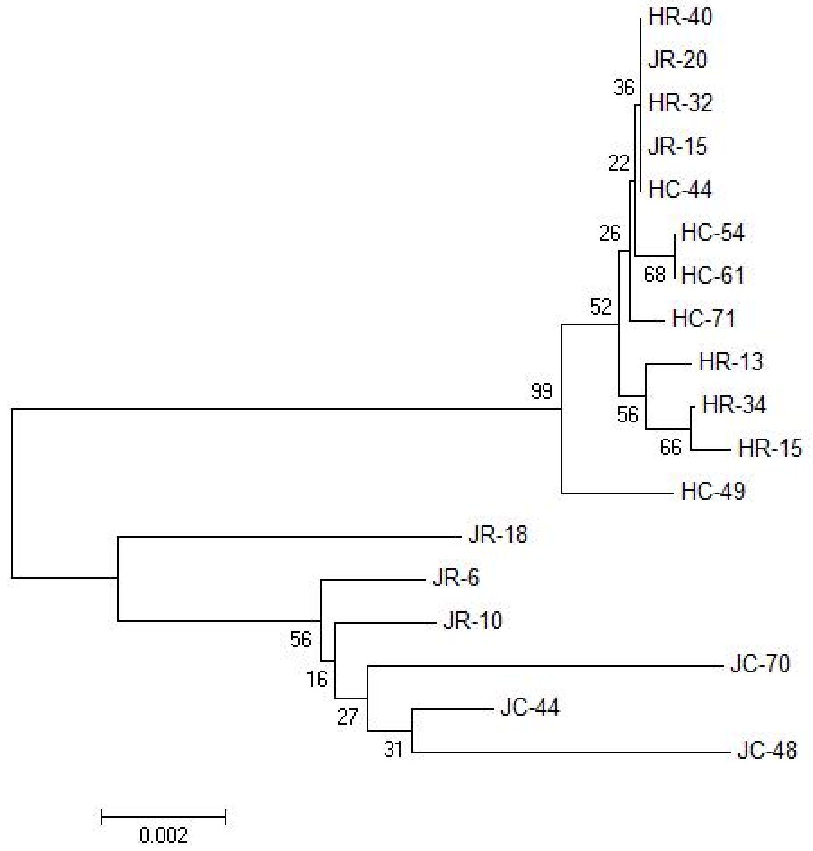

Figure S1. Phylogenetic tree analysis plot of 18 bacterial strains isolated from tilapia body and its living environment was carried out.

Table S1. Determination of volatile flavor compounds in fish flesh after storage for 0 and 6 days by GC-MS.

**No.**	**Compounds**	**Concentration（mg/L）**							
		**Control_0**	**HR_15_0**	**JR_18_0**	**HC_71_0**	**Control_6**	**HR_15_6**	**JR_18_6**	**HC_71_6**
1	Benzyl alcohol	0	0	0	0	0. 00600027	0	0	0
2	Phenylethyl alcohol	0	0	0. 00878002	0	0. 0570408	0. 0189116	0. 0337343	0. 0409916
3	1-Propanol,3-(methylthio)-	0	0	0	0	0. 00798234	0	0. 00408646	0. 0059424
4	1-Butanol	0	0	0. 026526	0	0	0	0	0
5	Isoamyl alcohol	0	0	0	0	0. 153931	0	0	0
6	n-Hexyl alcohol	0	0	0	0	0. 0199328	0	0. 0193308	0
7	2-Ethyl-1-hexanol	0. 0157037	0. 0242277	0. 020183	0. 0139456	0. 0240488	0. 0158369	0. 034609	0. 0103195
8	1-Heptanol, 6-Methyl-	0	0	0	0	0	0	0	0. 00618313
9	1-Octanol	0. 00889722	0	0. 0122797	0	0	0	0. 00863382	0

10	Cyclooctanol	0.0439114	0	0.0248942	0	0	0	0	0
11	1-Octen-3-ol	0.179814	0.0707772	0.200518	0.271222	0.314882	0.134928	0.131511	0.0798976
12	2-Octen-1-ol	0	0	0	0	0.0792827	0.0263469	0	0
13	cis-2-Nonen-1-ol	0	0	0	0	0.0716065	0	0	0
14	Ethyl-PEG4-alcohol	0	0.003824	0	0.00720218	0	0.0125542	0.0144037	0.00723195
15	Dichloromethane	0	0	0	0	0	0	0	0.00192791
16	Cyclodecane	0	0	0	0	0	0	0	0.0133213
17	naphthalene	0.00746916	0.00568929	0	0.0093948	0.0177583	0.0119786	0.0102225	0.0117814
18	1,3-Cyclooctadiene	0.0665207	0.0222672	0.0482567	0.0864693	0.166543	0.0521605	0.035915	0.0161463
19	1-Decene	0	0	0	0.0120163	0	0	0	0
20	2,5,5-Trimethyl-2-hexene	0	0	0.10486	0.145482	0	0.119025	0.0835227	0.0911434
21	Benzaldehyde	0.0059466	0.00543439	0.00850638	0.0136683	0.0574382	0.0331552	0.0412288	0.0265823
22	Phenylacetaldehyde	0	0	0	0	0	0	0.0127273	0
23	Hexanal	0.0019701	0	0	0	0	0	0	0
24	n-capryl(ic) aldehyde	0	0	0.0207473	0	0	0	0	0
25	Nonanal	0.0565697	0.0585565	0.0769311	0.0493641	0.0547983	0.0460169	0.0620162	0.0462601
26	Decanal	0.0306064	0.0326806	0.0192433	0.0145414	0	0	0	0
27	7-Methoxy-1,3-benzodioxole-5-carboxaldehyde	0	0	0	0	0.0163072	0	0	0.0229477
28	octadecanal	0	0	0	0	0	0.0151497	0	0
29	dimethylsilanediol	0	0	0	0.0197672	0	0.011725	0	0.0126715
30	Carbamic acid	0.106571	0	0.0916732	0	0	0.0368159	0	0.0260926
31	Ethyl 2-hydroxy-2-(4-hydroxyphenyl)acetate	0	0	0	0	0	0.01409	0	0
32	n-Butyl butanoate	0	0	0	0	0	0	0.0121213	0.00634573
33	Hexyl chlorocarbonate	0	0	0	0	0	0	0.019009	0
34	Formic acid, octylester	0	0	0.0130695	0	0	0	0	0
35	phenyl acetate	0.00381936	0	0	0	0	0	0	0
36	3-Octanone	0	0	0.011306	0	0	0	0	0.00680539
37	2-Methyl-3-Octanol	0	0	0	0.0145139	0	0	0	0
38	2,3-Octanedione	0	0	0	0	0.0136639	0	0	0

39	2,5-Octanedione	0	0	0	0	0	0	0.0138113	0
40	Phenol	0	0	0	0	0.0280136	0.00643652	0.0117643	0.00540673
41	2,4-Di-t-butylphenol	0.0846115	0.0637134	0.11579	0.0119234	0.159647	0.0823175	0.109517	0.0975851
42	Butylated hydroxytoluene	0.0123474	0.00954058	0.0227913	0.115976	0.0158433	0.0191552	0.00996138	0.0085268
43	biphenyl	0.00272173	0	0.00714821	0.00523731	0	0	0.00465877	0
44	Benzene,1,3-bis(1,1-dimethylethyl)-	0.430832	0.171806	0.628301	0.503079	0.27652	0.305435	0.210267	0.176572
